# Cross-cultural adaptation, validation, and measurement invariance of the First Aid Willingness Questionnaire (FAWQ) among Turkish adolescents

**DOI:** 10.1371/journal.pone.0361329

**Published:** 2026-10-23

**Authors:** Harun Özbey, Özlem Çalışır, Meral Bayat, Sultan Beşiktaş, Aklilu Abrham Roba

**Affiliations:** 1 Department of Child Health and Diseases Nursing, Faculty of Health Sciences, Erciyes University, Kayseri, Türkiye; 2 Department of Child Health and Diseases Nursing, Institute of Health Sciences, Erciyes University, Kayseri, Türkiye; 3 College of Health and Medical Sciences, Haramaya University, Dire Dawa, Ethiopia; IMU University, MALAYSIA

## Abstract

**Background:**

This study translated, culturally adapted, and evaluated the First Aid Willingness Questionnaire for Schoolchildren to assess adolescents’ self-reported readiness to help and potential barriers to first-aid provision.

**Methods:**

This cross-sectional psychometric study included 470 high school students in Aksaray, Türkiye. The questionnaire underwent forward–backward translation, review by 12 experts, and pilot testing with 35 students. Exploratory factor analysis derived the structure, which was subsequently evaluated via ordinal confirmatory factor analysis in the same sample. Content and construct validity, internal consistency, and measurement invariance across gender, grade level, and perceived family income were assessed.

**Results:**

All item-level content validity ratios exceeded 0.83; the scale-level content validity index was 0.90. Removing two items yielded a 16-item, three-factor structure: First Aid Willingness, Negative Emotions, and Perceived Knowledge. The model showed acceptable fit: scaled χ²(101) = 288.09, p < 0.001; comparative fit index = 0.95; Tucker–Lewis index = 0.94; root mean square error of approximation = 0.06, 90% confidence interval [0.05, 0.07]; standardized root mean square residual = 0.06. Composite reliability ranged from 0.79 to 0.90. Average variance extracted ranged from 0.49 to 0.59, with borderline convergent validity for Negative Emotions. Discriminant validity was supported. Cronbach’s alpha ranged from 0.72 to 0.87, and McDonald’s omega from 0.74 to 0.87. Configural, threshold, metric, and scalar invariance were supported across all grouping variables. Strict invariance was supported across grade level and perceived family income, but not gender.

**Conclusion:**

The results provide preliminary evidence for the Turkish questionnaire’s psychometric properties and support subscale interpretation and latent mean comparisons within the study sample. Independent replication and evaluation of temporal stability, responsiveness, and external validity are needed.

## Introduction

First aid can reduce harm following acute illness, injury, or other emergencies, but effective bystander assistance depends not only on possessing knowledge or skills but also on being willing and confident to intervene [1,2]. School-based first-aid education has been associated with improvements in adolescents’ knowledge, confidence, and willingness to help [3,4]. Willingness is therefore an important outcome to measure in its own right because an adolescent may know how to respond yet remain reluctant to act. Distinguishing willingness from emotional barriers and perceived knowledge may help identify where educational needs lie. Conceptually, willingness to provide first aid is multidimensional and may be influenced by attitudes, self-efficacy, perceived behavioral control, and situational factors. According to Ajzen’s Theory of Planned Behavior [5], behavioral intention is influenced by attitudes, subjective norms, and perceived behavioral control. Bandura’s self-efficacy framework similarly emphasizes belief in one’s capacity to act under challenging conditions [6], while the Bystander Intervention Model describes sequential cognitive processes involved in deciding whether to intervene [7]. Together, these perspectives provide a theoretical basis for examining cognitive, affective, and social dimensions of first-aid willingness.

In 2015, the World Health Organization (WHO) endorsed the “Kids Save Lives” declaration, which recommends annual cardiopulmonary resuscitation (CPR) training for children aged 12 years and older [8]. Subsequent studies have reported improvements in adolescents’ knowledge, confidence, and willingness to intervene following school-based training [9–13]. In Türkiye, certified first-aid training programs are regulated by the Ministry of Health through the First Aid Regulation [14]. National curriculum reform includes the Türkiye Yüzyılı Maarif Modeli, approved in 2024 [15]. With approximately 5.33 million students enrolled in upper-secondary education in the 2024–2025 academic year [16], the potential reach of school-based first-aid education is substantial. Given Türkiye’s vulnerability to natural disasters, a psychometrically supported instrument is needed to characterize adolescents’ first-aid willingness in this setting.

The First Aid Willingness Questionnaire for Schoolchildren (FAWQ) was developed in Hungary by Katona, Tarkó, and Berki [17] with 413 adolescents. Exploratory factor analysis identified a four-factor structure: first aid willingness for peers, first aid willingness for strangers, negative emotions (affective barriers such as fear and anxiety), and knowledge (perceived competence), with internal consistency values of α = 0.70–0.77 [17]. This structure is consistent with the theoretical frameworks presented above: the peer–stranger distinction reflects the attribution of social responsibility; the dimension of negative emotions captures affective barriers to intervention; and the knowledge dimension corresponds to perceived behavioral control and self-efficacy. A later study also found that the willingness to provide first aid was positively associated with prosocial behavior among Hungarian adolescents [18]. However, the FAWQ has not been cross-culturally adapted and psychometrically evaluated among Turkish adolescents, and it remains unclear whether the dimensional structure of the source instrument is retained in this context. This represents an important measurement gap because cross-cultural adaptation requires not only linguistic equivalence but also evidence that the underlying constructs are represented adequately and can be interpreted meaningfully in the target population. The present study therefore addresses two interrelated questions: whether a Turkish-language adaptation of the FAWQ provides an appropriate measure of adolescents’ self-reported first-aid willingness, negative emotions, and perceived knowledge, and whether the dimensional structure of the source instrument is reproduced in the present Turkish school sample. Its contribution thus extends beyond translation by providing a cross-cultural examination of the FAWQ’s internal structure, reliability, construct validity, and measurement comparability across relevant adolescent subgroups. Accordingly, the present study aimed to (a) translate and culturally adapt the FAWQ into Turkish, (b) examine its factor structure using exploratory factor analysis followed by ordinal confirmatory factor analysis, (c) assess convergent and discriminant validity, (d) evaluate internal-consistency reliability, and (e) test measurement invariance across gender, grade level, and perceived family income.

## Methods

### Study design and reporting

This cross-sectional psychometric study comprised two stages: (1) translation and cultural adaptation of the First Aid Willingness Questionnaire for Schoolchildren (FAWQ) into Turkish and (2) psychometric evaluation of the adapted instrument, including content validity, factor structure, construct validity, internal consistency, and measurement invariance. Reporting was guided by the Guidelines for Reporting Reliability and Agreement Studies (GRRAS) [19], while terminology concerning measurement properties and content validity was additionally aligned, where applicable, with contemporary COSMIN guidance [20].

### Translation and cultural adaptation

Translation and cultural adaptation followed a five-step forward–backward procedure [21–23]. Translators were selected based on proficiency in both English and Turkish and prior experience in linguistic translation. Two independent linguists first translated the source questionnaire from English into Turkish. The research team then reconciled the two forward translations, considering semantic, linguistic, idiomatic, conceptual, and contextual equivalence. Two additional independent linguists subsequently back-translated the reconciled Turkish version into English. The research team compared and synthesized the back-translations with the source instrument to produce the prefinal Turkish version.

Content validity was evaluated by a panel of 12 experts: six professors (PhD) and three associate professors (PhD) in Pediatric Nursing, two professors (MD) in Pediatrics, and one associate professor in Biostatistics. Experts were selected on the basis of academic expertise in child health, first-aid education, and measurement. Each expert rated every item for essentiality using Lawshe’s three-category format (essential / useful but not essential / not necessary) and for relevance. The item-level content validity ratio (CVR) was computed with Lawshe’s formula, CVR = (n_e_ − N/2)/(N/2), where n_e_ is the number of experts rating an item as essential and N is the total number of experts; for a 12-member panel the critical CVR is 0.56 [24]. The scale-level content validity index (S-CVI/Ave) was computed as the mean of the item-level relevance indices (I-CVI). All item-level CVRs exceeded 0.83 and the S-CVI/Ave was 0.90, exceeding the recommended thresholds and supporting the content validity of the Turkish FAWQ. Expert-panel composition and summary content-validity results are provided in S2 Table in S1 Appendix. The prefinal Turkish questionnaire was pilot-tested with 35 students recruited from an adjacent school that was not included in the main study. The pilot participants were comparable to the target population in terms of age range, grade level, and gender distribution, with approximately equal proportions of girls and boys. Each student completed the questionnaire and subsequently participated in an individual cognitive interview assessing the readability, clarity, comprehensibility, and potential ambiguity of each item. All items were understood as intended, and no participants suggested wording changes; consequently, no revisions were required before psychometric testing.

### Setting and participants

The study was conducted during the 2024–2025 academic year in high schools located in Aksaray, Türkiye. A convenience sampling strategy was used. Four high schools—two state and two private schools—were purposively selected from two central districts to maximize socioeconomic diversity, and all eligible students in the selected schools were invited to participate voluntarily.

Sample size was determined using multiple criteria. For the initial 18-item pool, the most conservative recommended ratio of 20 participants per item indicated a minimum sample of 360 students [25]. In addition, measurement-invariance testing requires adequate subgroup sizes, commonly at least 100–200 participants per group [26]. Accordingly, the target sample was 500 students. Across the four participating public and private high schools, the combined Grade 9 and Grade 10 enrolment was 1,038 students (School A = 241, School B = 304, School C = 212, School D = 281). Of these, 500 students expressed willingness to participate; 30 were excluded because written parental consent was not obtained, yielding a final analytic sample of 470 adolescents and an overall participation rate of 45.3% (109, 138, 96, and 127, respectively).

### Data collection and measures

Participants were recruited, and data were collected face to face from 1 to 30 November 2024 using the Student Information Form and the Turkish FAWQ. Eligible participants were adolescents aged 13–16 years who could understand, speak, read, and write Turkish; whose parents provided written consent; and who agreed to participate. The Student Information Form was a researcher-developed, nine-item questionnaire based on the relevant literature. It collected participant and parental sociodemographic characteristics and first-aid-related information [17,27].

The source FAWQ, developed by Katona, Tarkó, and Berki [17], comprised 18 items rated on a five-point response scale ranging from 1 (“not at all typical of me”) to 5 (“completely typical of me”). The original questionnaire proposed four domains: first-aid willingness toward peers, first-aid willingness toward strangers, negative emotions, and knowledge. Because the items in the knowledge domain assess adolescents’ self-reported knowledge or competence rather than performance on an objective knowledge test, the corresponding factor in the Turkish version is referred to as Perceived Knowledge. In the original study, subscale internal-consistency coefficients ranged from 0.70 to 0.77 [17]. The dimensionality and retained item set of the Turkish version were evaluated empirically in the present sample.

The full 18-item pool was translated here, retaining the source item numbering, so that item performance could be re-evaluated in the Turkish context without assuming the retention decisions of the source study, in which two items had been removed. S1 Table in S1 Appendix maps each Turkish item number to the corresponding source item number and content, the original and final factor assignments, and the retention status of each item. A later 20-item version of the FAWQ was subsequently published by Katona et al. (2024) [18]; the present study pertains specifically to the 2022 instrument and does not incorporate that later version. The final Turkish questionnaire and subscale scoring guide are provided in S1 Appendix. Subscale scores were calculated as the arithmetic mean of the constituent items on the original 1–5 response scale. Higher First Aid Willingness and Perceived Knowledge scores indicate greater readiness to act and self-assessed capability, respectively, whereas higher Negative Emotions scores indicate greater affective barriers. The subscales were interpreted separately, and no overall FAWQ total score was calculated.

### Ethics statement

Permission to translate and culturally adapt the FAWQ was obtained from the original questionnaire developer before the translation process. The study was conducted in accordance with the Declaration of Helsinki and was approved by the Erciyes University Social and Human Sciences Ethics Committee (26 March 2024; Approval No. 2024/103). Administrative permission was obtained from the participating institutions before data collection. Adolescents and their parents/guardians were informed about the study objectives and procedures. Written informed consent was obtained from parents/guardians and written assent was obtained from adolescents before participation.

### Statistical analysis

All statistical analyses were conducted in R version 4.4.1 using RStudio 2026.01.1 (Build 403; Posit Software, PBC). Data were imported from SPSS format using the haven package. Ordinal confirmatory factor analysis (CFA) was conducted using lavaan version 0.6–19, with the weighted least squares mean- and variance-adjusted (WLSMV) estimator, theta parameterization, standardized latent-variable identification, and all 16 items specified as ordered categorical indicators. Complete cases were analyzed, and no correlated item residuals were specified. Composite reliability and average variance extracted were calculated from the completely standardized factor loadings and residual variances, whereas maximum shared variance and the Fornell–Larcker criterion were derived from the latent-factor correlation matrix. Raw Cronbach’s alpha was calculated using the psych package. Model-based omega for the ordinal response scale, traditional heterotrait–monotrait (HTMT) ratios, and HTMT_2_ coefficients [28] were calculated using semTools version 0.5–7.

Participant characteristics were summarized using means and standard deviations for continuous variables and frequencies and percentages for categorical variables. No missing data were identified for any study variable, including the FAWQ items and grouping variables; therefore, all psychometric analyses used the full sample (N = 470). All tests were two-sided, with a significance level of 0.05; values below 0.001 were reported as *p < 0.001*. Content validity was evaluated using item-level CVRs and the scale-level CVI derived from the ratings of the 12 experts.

Exploratory factor analysis (EFA) was initially conducted on the 18 translated items using principal axis factoring of the polychoric correlation matrix, with Promax rotation (κ = 4) and Kaiser normalization. Sampling adequacy and factorability were evaluated using the Kaiser–Meyer–Olkin measure and Bartlett’s test of sphericity. Factor retention was evaluated using eigenvalues greater than 1.00, scree-plot inspection, polychoric parallel analysis, and theoretical interpretability. Parallel analysis was conducted on the initial 18-item polychoric correlation matrix using principal-axis factor extraction, 1,000 simulated datasets, and the 95th-percentile criterion. Because factor-retention criteria may yield different recommendations, the final number of retained factors was determined by considering the parallel-analysis results together with the EFA pattern structure, item communalities and cross-loadings, factor interpretability, reliability, and subsequent CFA diagnostics. Items were evaluated iteratively according to primary pattern coefficients, with 0.30 considered the minimum acceptable loading, extracted communalities, cross-loadings, and conceptual alignment with the emerging factors. The EFA was re-estimated after each item removal. Because an independent validation sample was not available, EFA and CFA were conducted sequentially in the full sample (*N* = 470).

The retained 16-item, three-factor structure was evaluated using ordinal confirmatory factor analysis in R with WLSMV estimation and theta parameterization. All FAWQ items were specified as ordered categorical indicators. Each item loaded only on its intended factor, the three latent factors were allowed to correlate, and no residual covariances were specified. Items 17 (“I would be afraid to help someone who is unconscious”) and 18 (“In the case of a serious accident in my neighbourhood, I would panic”) were both Negative Emotions items in the source instrument, assessing anticipated fear and panic. In the initial 18-item EFA, these two items formed a separate, weak fourth dimension; when three factors were extracted, Item 18 showed no loading ≥ 0.30 on any factor and Item 17 loaded −0.39 on the third factor. After Item 17 was removed because of its cross-loading pattern, Item 18 continued to show insufficient communality. Although expert review had judged all translated items essential and relevant, these two items were removed on combined empirical grounds; their removal did not eliminate the Negative Emotions construct, which remained represented by four items with acceptable reliability, but the deletions are acknowledged as data-driven and requiring confirmation in an independent sample.

Model fit was evaluated using the chi-square statistic, chi-square-to-degrees-of-freedom ratio, comparative fit index (CFI), Tucker–Lewis index (TLI), root mean square error of approximation (RMSEA) with its 90% confidence interval, and standardized root mean square residual (SRMR). Model fit was interpreted holistically. CFI and TLI values of at least 0.90 and RMSEA and SRMR values no greater than 0.08 were considered acceptable; values approaching CFI/TLI ≥ 0.95, RMSEA ≤ 0.06, and SRMR ≤ 0.08 indicated close fit [26,29].

Convergent validity was assessed using standardized factor loadings, composite reliability (CR), and average variance extracted (AVE). CR values of at least 0.70 and AVE values of at least 0.50 were used as reference criteria [30]. Discriminant validity was evaluated using maximum shared variance (MSV < AVE), the Fornell–Larcker criterion [30], and heterotrait–monotrait ratios (HTMT and HTMT_2_ < 0.85) [31]. Traditional pairwise HTMT coefficients were reported in matrix form, whereas the maximum HTMT_2_ coefficient was used as the construct-level summary.

Internal consistency was assessed using Cronbach’s alpha and McDonald’s omega for each subscale, corrected item–total correlations, and alpha if item deleted. Omega and alpha values of at least 0.70 were used as conventional reference values [32,33], and corrected item–total correlations of at least 0.30 were considered acceptable [34]. Reliability was evaluated and interpreted separately for the three supported subscales. Because the present measurement model did not test a general or higher-order FAWQ factor, the overall total score was not interpreted as a psychometrically validated score.

Measurement invariance was examined across gender, grade level, and perceived family income because these characteristics are theoretically and empirically associated with adolescents’ first-aid attitudes, willingness, and self-appraised competence, and because meaningful comparison of these groups requires prior evidence that the instrument measures the same constructs in the same way across them. Previous first-aid training and experience were used for exploratory known-groups comparisons; measurement invariance across these groups was not examined. Measurement invariance of the final ordinal three-factor model was evaluated across gender (male, n = 217; female, n = 253), grade level (Grade 9, n = 169; Grade 10, n = 301), and perceived family income. Multigroup CFA was estimated using WLSMV with theta parameterization. Equality-constrained models were generated using semTools::measEq.syntax() with standardized latent-variable identification (ID.fac = “std.lv”) and the Wu–Estabrook identification method for ordered categorical indicators (ID.cat = “Wu.Estabrook.2016”). Five models were evaluated sequentially: configural invariance, with no cross-group equality constraints; threshold invariance, with item thresholds constrained equal across groups; metric invariance, with thresholds and factor loadings constrained equal; scalar invariance, with additional equality constraints on intercept-related parameters under the ordinal identification scheme; and strict invariance, with residual variances additionally constrained equal. Scalar invariance was considered sufficient for comparison of latent factor means, whereas strict invariance was examined as an additional test of residual-variance equality and was not required for latent-mean comparability [35]. Invariance was supported when ΔCFI ≥ −0.010 and |ΔRMSEA| ≤ 0.020 [36]. Perceived family income was self-reported as poor, middle, or good. Because only 11 participants reported poor perceived family income, the poor and middle categories were combined to avoid estimation based on an inadequately small subgroup, resulting in a poor/middle group (n = 209) that was compared with the good-income group (n = 261). Accordingly, income-related invariance results refer specifically to the poor/middle versus good perceived-family-income comparison. Because categorical-robust CFI and RMSEA corrections were unavailable for at least one multigroup model, scaled WLSMV CFI and RMSEA values were used consistently across the measurement-invariance analyses.

## Results

### Sociodemographic characteristics

The study included 470 adolescents with a mean age of 14.71 ± 0.62 years. Of the participants, 253 (53.83%) were female, 301 (64.04%) were in the 10th grade, and 415 (88.30%) lived in nuclear families. More than half reported good family income (n = 261, 55.53%). Most participants had never provided first aid to an injured person (n = 299, 63.62%) and had not received first aid training (n = 324, 68.94%). Nevertheless, nearly all considered first-aid knowledge necessary (n = 449, 95.53%), and 406 (86.38%) believed that first-aid training should be provided in primary school (Table 1).

**Table 1:** Sociodemographic and first-aid-related characteristics of participants (N = 470).

Characteristic	Category	Frequency	%
Gender	Female	253	53.83
	Male	217	46.17
Grade level	Grade 9	169	35.96
	Grade 10	301	64.04
Family type	Nuclear family	415	88.3
	Extended family	30	6.38
	Disrupted family	25	5.32
Perceived family income	Poor	11	2.34
	Middle	198	42.13
	Good	261	55.53
Experience of providing first aid to an injured person	Yes	171	36.38
	No	299	63.62
Previous receipt of first-aid training	Yes	146	31.06
	No	324	68.94
Perceived necessity of first-aid knowledge	Yes	449	95.53
	No	21	4.47
Support for providing first-aid training in primary school	Yes	406	86.38
	No	64	13.62

*Note:* Family structure was classified into three categories: Nuclear family (parents and children living together), Extended family (nuclear family plus other relatives sharing the household), and Disrupted family (households affected by parental divorce, separation, or death).

Item-level response distributions and univariate skewness and kurtosis for all items are reported in S3 Table in S1 Appendix. All items used the full 1–5 response range, with no item showing floor or ceiling concentration that would preclude ordinal factor analysis.

### Content validity

Content validity was evaluated by 12 experts. All item-level content validity ratio values exceeded 0.83, and the scale-level content validity index was 0.90, demonstrating strong expert agreement regarding the essentiality and relevance of the translated items and supporting the content validity of the Turkish FAWQ.

### Construct validity

#### Exploratory factor analysis.

Exploratory factor analysis was initially conducted on the 18-item FAWQ using principal axis factoring based on the polychoric correlation matrix, with Promax rotation and Kaiser normalization. Polychoric parallel analysis of the initial 18-item pool recommended four factors. The first four observed factor eigenvalues (4.814, 1.624, 1.184, and 0.512) exceeded the corresponding 95th-percentile eigenvalues from the simulated datasets (0.44, 0.30, 0.25, and 0.21, respectively), whereas the fifth observed eigenvalue did not exceed the simulated criterion (0.169 vs. 0.170). Thus, parallel analysis indicated four non-random statistical dimensions in the initial 18-item data.

Consistent with this result, the initial extraction produced four factors. However, examination of the factor pattern showed that the fourth dimension was defined only by Items 17 and 18. Item 17 showed cross-loadings across Factors 2, 3, and 4 (−0.31, 0.32, and 0.40, respectively), preventing its unambiguous assignment to a single factor. Item 17 was therefore removed, and the analysis was repeated with the remaining 17 items. In the subsequent 17-item solution, Item 18 exhibited an unacceptably low initial communality of 0.09 and an extracted communality of only 0.11, failing to meet acceptable psychometric thresholds for shared variance. Consequently, Item 18 was removed due to insufficient communality, and the final analysis was rerun using the remaining 16 items.

The final 16-item solution demonstrated good factorability, with a Kaiser–Meyer–Olkin measure of 0.87 and a significant Bartlett’s test of sphericity, χ^2^(120) = 3260.42, p < 0.001. Three factors were retained, with initial eigenvalues of 5.33, 2.47, and 1.92; the fourth eigenvalue was below 1.00 (0.89). The resulting structure comprised First Aid Willingness (nine items), Negative Emotions (four items), and Perceived Knowledge (three items). Pattern coefficients ranged from 0.57 to 0.86, with no cross-loadings of 0.30 or greater, and extracted communalities ranged from 0.31 to 0.69. The three factors accounted for 60.72% of the total variance based on the initial eigenvalues and 52.26% after extraction. The factor-specific extracted variance was 30.38% for First Aid Willingness, 12.40% for Negative Emotions, and 9.48% for Perceived Knowledge (Table 2). Interfactor correlations were modest, ranging from 0.15 to 0.33, supporting a coherent and interpretable three-factor structure for the Turkish FAWQ.

**Table 2:** Exploratory factor analysis of FAWQ.

Subscale	Item	Pattern coefficient	h²	Initial eigenvalue (λ)	Initial variance (%)	Extracted variance (%)
First Aid Willingness	I13	0.80	0.65	5.33	33.28	30.38
	I11	0.79	0.59			
	I14	0.77	0.60			
	I16	0.73	0.61			
	I1	0.72	0.45			
	I4	0.71	0.54			
	I9	0.59	0.40			
	I5	0.58	0.31			
	I8	0.57	0.44			
Negative Emotions	I12	0.84	0.69	2.47	15.43	12.40
	I6	0.66	0.45			
	I7	0.64	0.41			
	I3	0.64	0.41			
Perceived Knowledge	I10	0.86	0.68	1.92	12.01	9.48
	I2	0.78	0.60			
	I15	0.66	0.54			

*Note. N = 470. Extraction was performed using principal axis factoring based on the polychoric correlation matrix, followed by Promax rotation with Kaiser normalization. Values in the pattern-coefficient column are standardized primary pattern coefficients; no secondary coefficient reached an absolute value of 0.30. h^2^ = extracted communality; λ = initial eigenvalue. Initial eigenvalues and initial variance percentages were derived from the unreduced polychoric correlation matrix. Extracted variance percentages were derived from the unrotated principal-axis solution. The first three initial eigenvalues accounted for 60.72% of the total variance, and the three-factor extraction accounted for 52.26%.*

### Confirmatory factor analysis

Ordinal confirmatory factor analysis supported the final 16-item, three-factor model. Although the scaled chi-square test was significant, scaled χ^2^(101) = 288.09, p < 0.001, the relative chi-square was acceptable (χ^2^/df = 2.85). The scaled fit indices indicated acceptable-to-good model fit: CFI = 0.95, TLI = 0.94, RMSEA = 0.06, 90% CI [0.05, 0.07], and SRMR = 0.06. Standardized factor loadings ranged from 0.54 to 0.89, and latent-factor correlations ranged from 0.14 to 0.38.

To evaluate whether the merged First Aid Willingness factor was preferable to the source structure that distinguished peer- from stranger-directed willingness, we fitted a competing four-factor model to the same 16 retained items using ordinal WLSMV estimation with theta parameterization. The four-factor model showed poor fit, scaled χ^2^(98) = 1059.13, p < 0.001; CFI = 0.76; TLI = 0.70; RMSEA = 0.14, 90% CI [0.14, 0.15]; SRMR = 0.11, and yielded an estimated correlation of 0.94 between the peer and stranger latent factors, indicating that the two dimensions were very highly correlated in the present sample. By contrast, the three-factor model with a single First Aid Willingness factor fitted the same items well (scaled χ^2^(101) = 288.09; CFI = 0.95; TLI = 0.94; RMSEA = 0.06, 90% CI [0.05, 0.07]; SRMR = 0.06). The three-factor specification showed substantially better fit than the four-factor specification in the present sample. Because the 18-item and 16-item models contain different item sets, no chi-square difference test between them was conducted; instead, the two competing specifications were compared on the identical 16-item set.

The complete pattern matrices and communalities for the initial 18-item and intermediate 17-item solutions are provided in S4 and S5 Tables in S1 Appendix, and the standardized loadings of the final three-factor model are reported in S6 Table in S1 Appendix.

Collectively, these findings supported the three-factor structure of the Turkish FAWQ (Fig 1).

**Fig 1:**
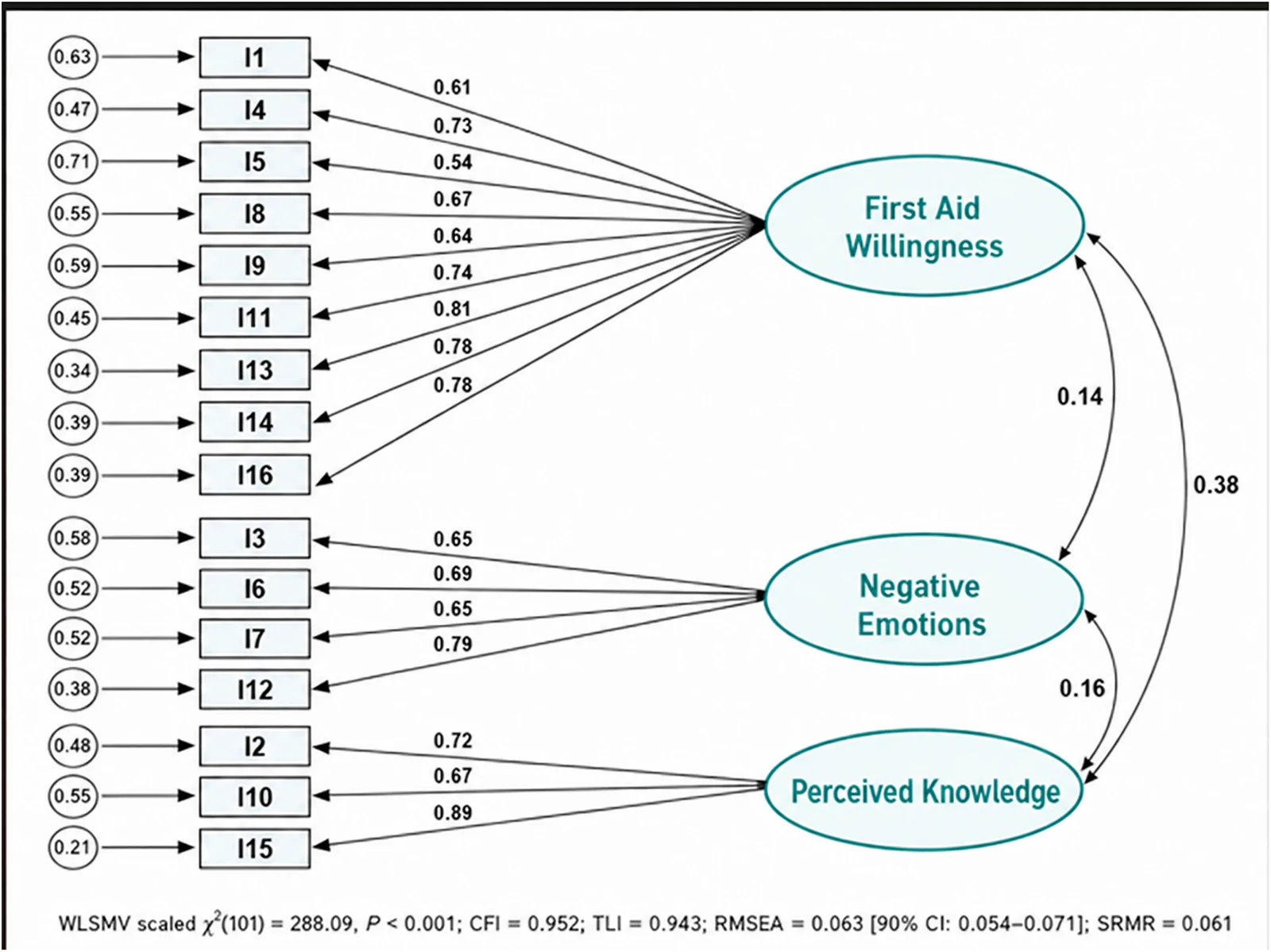
Confirmatory factor analysis model with standardized factor loadings.

### Convergent and discriminant validity

Composite reliability ranged from 0.79 to 0.90, exceeding the recommended 0.70 criterion for all three factors. AVE values ranged from 0.49 to 0.59, with Perceived Knowledge exceeding the 0.50 threshold, First Aid Willingness meeting it, and Negative Emotions falling only marginally below it. Considered together with the strong composite-reliability estimates, these findings provided preliminary convergent-validity evidence, with borderline support for Negative Emotions [30]. Discriminant validity was also supported across all factors: MSV values ranged from 0.02 to 0.14 and remained lower than the corresponding AVE estimates, while maximum HTMT_2_ values ranged from 0.13 to 0.31, well below the conservative 0.85 threshold. Internal-consistency reliability was acceptable to good, with Cronbach’s α ranging from 0.72 to 0.87 and McDonald’s ω ranging from 0.74 to 0.87 (Table 3).

**Table 3:** Convergent validity, discriminant validity, and internal-consistency reliability of the three-factor Turkish FAWQ.

Construct	CR	AVE	MSV	Cronbach’s alpha	Maximum HTMT_2_	McDonald’s omega
First Aid Willingness	0.90	0.50	0.14	0.87	0.31	0.87
Negative Emotions	0.79	0.49	0.02	0.73	0.13	0.74
Perceived Knowledge	0.81	0.59	0.14	0.72	0.31	0.77

The Fornell–Larcker criterion further supported discriminant validity among the three Turkish FAWQ factors. The square roots of AVE on the diagonal were 0.71 for First Aid Willingness, 0.70 for Negative Emotions, and 0.77 for Perceived Knowledge, and each exceeded the corresponding interfactor correlations, which ranged from 0.14 to 0.38. These findings indicate that each factor shared more variance with its own indicators than with the other factors, supporting the distinctiveness of the three constructs (Table 4).

**Table 4:** Fornell–Larcker criterion for discriminant validity of the three-factor Turkish FAWQ.

Construct	Factor 1	Factor 2	Factor 3
Factor 1: First Aid Willingness	0.71		
Factor 2: Negative Emotions	0.14	0.70	
Factor 3: Perceived Knowledge	0.38	0.16	0.77

Note. Diagonal values are the square roots of average variance extracted; values below the diagonal are latent-factor correlations. The upper triangle is omitted.

The HTMT results provided further evidence of discriminant validity among the three Turkish FAWQ factors. Pairwise HTMT values ranged from 0.15 to 0.35, with the highest coefficient observed between First Aid Willingness and Perceived Knowledge. All values were well below the conservative threshold of 0.85, supporting the empirical distinctiveness of the three factors (Table 5).

**Table 5:** Heterotrait–monotrait ratio matrix for discriminant validity of the three-factor Turkish FAWQ.

Construct	Factor 1	Factor 2	Factor 3
Factor 1: First Aid Willingness	1		
Factor 2: Negative Emotions	0.15	1	
Factor 3: Perceived Knowledge	0.35	0.15	1

**Note.** HTMT = heterotrait–monotrait ratio. Values below the diagonal represent pairwise HTMT coefficients, and diagonal values were fixed at 1.00. HTMT values below 0.85 indicate adequate discriminant validity. This table reports traditional HTMT coefficients, whereas the maximum HTMT_2_ values are presented in Table 3.

### Reliability analysis

All three FAWQ subscales demonstrated acceptable to good internal consistency. Corrected item–total correlations ranged from 0.46 to 0.70, with all values exceeding the conventional 0.30 criterion. Cronbach’s α was 0.87 for First Aid Willingness, 0.73 for Negative Emotions, and 0.72 for Perceived Knowledge. Deleting individual items did not increase the α coefficient of any subscale, supporting retention of all 16 items (Table 6).

**Table 6:** Reliability results for the three-factor FAWQ.

Subscale	Item	Cronbach’s α	Corrected item–total correlation	α if item deleted
First Aid Willingness	I13	*α = 0.87*	0.70	0.84
	I11		0.65	0.85
	I14		0.66	0.84
	I16		0.67	0.84
	I4		0.62	0.85
	I1		0.54	0.86
	I9		0.53	0.86
	I5		0.46	0.86
	I8		0.54	0.86
Negative Emotions	I12	*α = 0.73*	0.62	0.63
	I6		0.51	0.69
	I7		0.50	0.69
	I3		0.50	0.69
Perceived Knowledge	I2	*α = 0.72*	0.59	0.58
	I10		0.55	0.63
	I15		0.51	0.70

### Known-groups validity

As exploratory known-groups evidence, subscale scores were compared between students with versus without previous first-aid training and between those with versus without previous experience of providing first aid, using Welch’s t-tests (corroborated by Mann–Whitney U tests) and Hedges’ g with 95% confidence intervals. Students with previous training reported significantly higher Perceived Knowledge (g = 0.56, 95% CI [0.36, 0.76]) but did not differ in First Aid Willingness or Negative Emotions. Students with previous experience of providing first aid reported higher Perceived Knowledge (g = 0.64, 95% CI [0.45, 0.83]) and modestly higher First Aid Willingness (g = 0.22, 95% CI [0.03, 0.40]), with no difference in Negative Emotions. These associations are consistent with the expected direction and provide preliminary known-groups support for the construct interpretation of the subscales. As the design was cross-sectional, these differences are associational and must not be interpreted causally.

### Measurement invariance

Multigroup WLSMV analyses showed acceptable configural fit across gender (CFI = 0.945, RMSEA = 0.069, SRMR = 0.076), grade level (CFI = 0.956, RMSEA = 0.061, SRMR = 0.068), and perceived family income (CFI = 0.944, RMSEA = 0.069, SRMR = 0.074). As a sensitivity analysis, the 11 participants reporting poor perceived family income were excluded and the invariance sequence was re-estimated comparing only middle- (n = 198) and good-income (n = 261) groups; configural, threshold, metric, scalar, and strict invariance remained supported (all ΔCFI ≥ −0.003 and |ΔRMSEA| ≤ 0.003), indicating that the income-invariance conclusion was not dependent on the small poor-income subgroup. Threshold, metric, and scalar invariance were supported across all three grouping variables, with sequential ΔCFI values ranging from −0.003 to 0.001 and ΔRMSEA values ranging from −0.004 to −0.001, all within the prespecified criteria of ΔCFI ≥ −0.010 and |ΔRMSEA| ≤ 0.020. Strict invariance was also supported across grade level (ΔCFI = 0.000; ΔRMSEA = −0.002) and perceived family income (ΔCFI = 0.001; ΔRMSEA = −0.003), but not across gender, where ΔCFI decreased to −0.012 despite an acceptable ΔRMSEA of 0.004 (Table 7). Within the present sample, scalar invariance supports comparison of latent factor means across gender, grade level, and poor/middle versus good perceived family income. Strict invariance was not supported across gender, indicating that complete equality of residual variances across gender was not established.

**Table 7:** Measurement invariance of the three-factor Turkish FAWQ across gender, grade level, and perceived family income.

Group	Model	χ²	df	CFI	RMSEA	SRMR	ΔCFI	ΔRMSEA
Gender	Configural	426.83	202	0.945	0.069	0.076	—	—
	Threshold	468.64	234	0.943	0.065	0.076	−0.002	−0.004
	Metric	489.04	247	0.941	0.065	0.076	−0.002	−0.001
	Scalar	510.26	260	0.939	0.064	0.077	−0.002	−0.001
	Strict	575.83	276	0.927	0.068	0.082	−0.012	0.004
Grade level	Configural	379.40	202	0.956	0.061	0.068	—	—
	Threshold	421.30	234	0.954	0.058	0.068	−0.002	−0.003
	Metric	438.24	247	0.953	0.058	0.069	−0.001	−0.001
	Scalar	445.75	260	0.954	0.055	0.069	0.001	−0.002
	Strict	460.05	276	0.954	0.053	0.071	0.000	−0.002
Perceived family income	Configural	427.18	202	0.944	0.069	0.074	—	—
	Threshold	472.35	234	0.940	0.066	0.074	−0.003	−0.003
	Metric	489.07	247	0.940	0.065	0.075	−0.001	−0.001
	Scalar	500.97	260	0.940	0.063	0.076	0.000	−0.002
	Strict	511.51	276	0.941	0.060	0.078	0.001	−0.003

**Note.** The FAWQ items were treated as ordered categorical indicators. χ² values are WLSMV scaled-shifted chi-square statistics; CFI and RMSEA are WLSMV scaled fit indices. SRMR = standardized root mean square residual. Threshold invariance was tested explicitly because the indicators were ordinal. ΔCFI and ΔRMSEA represent sequential changes from the immediately preceding model: threshold versus configural, metric versus threshold, scalar versus metric, and strict versus scalar. Invariance was supported when ΔCFI ≥ −0.010 and |ΔRMSEA| ≤ 0.020. Gender groups were male (n = 217) and female (n = 253); grade-level groups were Grade 9 (n = 169) and Grade 10 (n = 301); perceived-family-income groups were poor/middle income (n = 209) and good income (n = 261).

## Discussion

The Turkish FAWQ demonstrated a coherent 16-item, three-factor structure with satisfactory validity, reliability, and broad measurement equivalence across the examined adolescent groups. Expert review established its content validity, EFA and ordinal CFA supported the domains of First Aid Willingness, Negative Emotions, and Perceived Knowledge, and complementary coefficients indicated acceptable-to-good internal consistency alongside clear discriminant validity. Moreover, scalar invariance across gender, grade level, and perceived family income provides a sound basis for comparing latent factor means among these groups.

Content validity was strong in this study. All item-level CVRs exceeded 0.83, and the scale-level CVI reached 0.90. These findings indicate strong expert agreement that the translated items were essential, relevant, and representative of the intended construct. Content validity provides the foundation for interpreting subsequent psychometric findings because even strong statistical performance cannot compensate for items that inadequately represent the construct. The translation, expert-review, and pilot-testing procedures collectively addressed key aspects of relevance and comprehensibility emphasized in contemporary guidance [20].

In addition to the content-validity evidence, exploratory factor analysis identified a three-factor structure comprising First Aid Willingness, Negative Emotions, and Perceived Knowledge. Item 17 was removed because of problematic cross-loadings, whereas Item 18 was excluded because of insufficient communality, yielding a more coherent 16-item solution. Parallel analysis of the original 18-item pool, however, indicated four non-random dimensions. This apparent discrepancy was examined in conjunction with the item-level results: the fourth dimension was defined solely by Items 17 and 18 and lacked adequate psychometric stability and substantive interpretability, given the cross-loading of Item 17 and the poor communality of Item 18 after re-estimation. The retained three-factor structure therefore reflects converging evidence from parallel analysis, factor-pattern structure, item-level diagnostics, reliability, theoretical interpretability, and subsequent CFA findings rather than reliance on a single retention criterion. Nevertheless, because the three-factor model was derived and evaluated within the same sample, independent replication is required to establish its structural stability and generalizability.

Whereas the original Hungarian instrument [17] distinguished willingness to help peers from willingness to help strangers, these items converged into a single, broader First Aid Willingness factor in the present Turkish sample. This structural difference is noteworthy but cannot be attributed specifically to cultural influences on the basis of the present data. Cultural context may be one explanation, but translation effects, item functioning, age and grade composition, sampling characteristics, and sample-specific variation remain plausible alternatives. A further divergence involved source Items 6 and 12, which describe immediately helping a brother after a bicycle accident or a fall while skating. These were classified as peer-directed willingness items in the source instrument but loaded on the Negative Emotions factor in the Turkish sample. These scenarios may have elicited stronger affective responses to the injury of a close family member than generalized willingness to intervene; however, alternative explanations, including translation nuances, item-specific content, and sample-dependent response patterns, should also be considered. Independent replication and direct cross-cultural comparison are therefore required before these structural differences can be interpreted as culturally specific.

Ordinal CFA provided additional in-sample evidence regarding the fit of the EFA-derived three-factor specification. The observed fit indices (CFI = 0.95, TLI = 0.94, RMSEA = 0.06, 90% CI [0.05, 0.07], and SRMR = 0.06), together with standardized factor loadings ranging from 0.54 to 0.89, were compatible with the proposed structure. The modest correlations among First Aid Willingness, Negative Emotions, and Perceived Knowledge further indicated that these domains were related but empirically distinguishable within the present sample. However, because the CFA was conducted in the same participants used to derive the structure through EFA, these findings should be interpreted as an internal evaluation of the proposed model rather than as independent structural confirmation. The three-factor structure therefore remains provisional pending replication in an independent sample.

Convergent-validity evidence for the Turkish FAWQ was encouraging and supported the integrity of the three-factor measurement model. Composite reliability estimates ranged from 0.79 to 0.90, exceeding the conventional 0.70 criterion for all factors and demonstrating satisfactory internal consistency of the items representing their respective latent constructs [32]. Although the AVE value for Negative Emotions (0.49) was modestly below the conservative 0.50 benchmark, the remaining domains reached or exceeded it (0.50 for First Aid Willingness and 0.59 for Perceived Knowledge), which should be interpreted alongside the strong composite reliability estimates. Under the Fornell–Larcker framework, a construct may retain acceptable convergent validity when AVE is slightly below 0.50 but composite reliability exceeds 0.60 [30]. Collectively, these findings indicate that the Turkish FAWQ factors are coherent and provide promising preliminary within-sample evidence, offering a basis for future studies linking FAWQ scores with objectively assessed first-aid knowledge, situational confidence, practical competence, and real-world helping behavior.

Multiple discriminant-validity indices indicated that First Aid Willingness, Negative Emotions, and Perceived Knowledge were empirically distinguishable within the present sample. This distinction is also conceptually meaningful: First Aid Willingness reflects readiness to act, Negative Emotions captures affective barriers that may inhibit intervention, and Perceived Knowledge represents self-assessed capability rather than objectively demonstrated competence [17]. Accordingly, low willingness to provide first aid may arise through different mechanisms; for example, an adolescent may perceive adequate knowledge yet still report anxiety, fear of making an error, or hesitation about intervening. Conversely, willingness to help does not necessarily imply sufficient factual knowledge or practical competence. Because objective first-aid competence and actual helping behavior were not assessed, these interpretations should remain confined to the self-reported domains measured by the FAWQ.

Internal consistency was acceptable to good across all three subscales based on conventional psychometric thresholds [32,37]. Cronbach’s alpha ranged from 0.72 to 0.87, McDonald’s omega ranged from 0.74 to 0.87, and corrected item–total correlations ranged from 0.46 to 0.70, all exceeding the conventional 0.30 benchmark for item retention [34]. The close agreement between alpha and omega, which are based on different measurement assumptions, provides convergent evidence for the internal consistency of the three subscales [33]. In addition, deletion of individual items did not improve subscale reliability, supporting retention of the final 16-item set. Further research should evaluate test–retest reliability to determine the temporal stability of the FAWQ subscale scores under conditions in which no meaningful change in first-aid readiness is expected. Measurement-invariance analyses provided strong evidence that the Turkish FAWQ functions comparably across gender, grade level, and perceived family income. Configural, threshold, metric, and scalar invariance were supported across all groups, indicating that adolescents organize the FAWQ constructs similarly and that the items relate to the underlying factors in a comparable manner. Crucially, scalar invariance provides a robust psychometric basis for comparing latent factor means across these demographic groups when using ordered-polytomous indicators [35,38]. Strict invariance was additionally supported across grade level and perceived family income, providing further evidence of residual-variance equality for these groups.

A key strength of this study lies in the multimethod validation strategy applied to the Turkish FAWQ, encompassing forward–backward translation, expert appraisal, pilot testing, exploratory and confirmatory factor analyses, convergent and discriminant validity assessments, complementary reliability coefficients, and ordinal multigroup invariance testing. This integrated approach provides a coherent body of evidence for the instrument’s cultural suitability, internal structure, reliability, and comparability across key adolescent groups.

## Limitations

This study has several limitations. First, EFA and CFA were conducted sequentially in the same sample; thus, the CFA represents an in-sample evaluation of the EFA-derived structure rather than an independent confirmation. Replication in an independent sample is therefore required. Second, participants were recruited by convenience from four purposively selected schools in Aksaray, with representation limited to Grades 9 and 10. Students were consequently clustered within a small number of schools, yet school-level clustering was not modeled; moreover, the participation of only four schools limited the feasibility of reliable cluster-level adjustment. The findings should therefore be considered sample-specific and generalized cautiously beyond the study setting, particularly to adolescents from rural areas, other geographical regions, different grade or age groups, or other cultural and linguistic contexts. Third, the cross-sectional design precluded evaluation of test–retest reliability and responsiveness to change. Finally, criterion-related validity, objective first-aid knowledge and practical competence, simulated or observed helping behavior, and predictive validity were not assessed. Measurement invariance across previous first-aid training and experience groups was not examined, so the exploratory known-groups comparisons require cautious interpretation. Future multicenter, longitudinal, intervention-based, and criterion-based studies using independent samples are needed to further establish the structural stability, temporal reliability, responsiveness, and external validity of the Turkish FAWQ.

## Implications for practice

Within the scope of the evidence generated in this cross-sectional study, the Turkish FAWQ may be useful for characterizing three self-reported domains—First Aid Willingness, Negative Emotions, and Perceived Knowledge—in school-health research and needs assessment. The subscale profiles may help researchers distinguish motivational readiness, emotional barriers, and perceived knowledge when describing adolescent first-aid preparedness. However, responsiveness to educational change was not evaluated; use of the instrument as an outcome measure in intervention studies should therefore be considered provisional until longitudinal responsiveness has been demonstrated. FAWQ scores should likewise not be interpreted as measures of objective first-aid competence or as predictors of actual helping behavior on the basis of the present study.

## Conclusions

This study advances the cross-cultural evaluation of the FAWQ by identifying a 16-item, three-factor Turkish structure comprising First Aid Willingness, Negative Emotions, and Perceived Knowledge among adolescents attending selected high schools in Aksaray. The adapted instrument demonstrated satisfactory content validity, an interpretable internal structure, acceptable-to-good subscale reliability, and clear discriminant validity, while convergent validity for Negative Emotions was borderline. Evidence of scalar invariance further supports comparison of latent factor means across gender, grade level, and poor/middle versus good perceived-family-income groups within the present sample. Taken together, these findings support interpretation of the Turkish FAWQ at the subscale level and indicate its potential value for examining distinct dimensions of adolescents’ first-aid readiness in school-health research. Because the three-factor structure was derived and evaluated in the same sample, independent replication is required before the structure can be considered firmly established. Future studies should also evaluate temporal stability, responsiveness, and associations with objective first-aid competence and helping behavior.

## Supporting information

S1 Appendix Supplementary tables, the final questionnaire, and scoring information for the Turkish FAWQ. Contains Table S1 (item mapping), Table S2 (expert-panel composition and summary content-validity results), Table S3 (item-response distributions), Tables S4 and S5 (initial and intermediate EFA pattern matrices), Table S6 (final CFA loadings), the final 16-item Turkish questionnaire, and the subscale scoring guide.
(DOCX)

S1 Dataset The de-identified participant-level dataset underlying the reported analyses. (CSV)

S1 Questionnaire The Turkish-language student information form and the 18-item FAWQ questionnaire used for data collection. (DOCX)
