# Supplementary material for: Cross-cultural adaptation, validation, and measurement invariance of the First Aid Willingness Questionnaire (FAWQ) among Turkish adolescents
Source: PLoS One. 2026 Oct 23;21(10):e0361329. doi: 10.1371/journal.pone.0361329 (PMC13693896; doi:10.1371/journal.pone.0361329)
Supplement: Supplementary file 1 [file pone.0361329.s001.docx]

**Supplementary Materials**

*Cross-Cultural Adaptation, Validation, and Measurement Invariance of the First Aid Willingness Questionnaire (FAWQ) Among Turkish Adolescents*

**Table S1. Item-mapping between the source and Turkish FAWQ**

| **Item** | **Content (English)** | **Source factor** | **Final Turkish factor** | **Status** |
| --- | --- | --- | --- | --- |
| 1 | My friend is injured on the playground. I help him immediately. | Peer willingness | First Aid Willingness | Retained |
| 2 | I see a bleeding head. I know how to apply a pressure bandage. | Knowledge | Perceived Knowledge | Retained |
| 3 | My classmate cut his finger and it bled. I became anxious. | Negative Emotions | Negative Emotions | Retained |
| 4 | An old man faints in the street. I notice him and call for help. | Stranger willingness | First Aid Willingness | Retained |
| 5 | My cousin seems to break his arm. I help him immediately. | Peer willingness | First Aid Willingness | Retained |
| 6 | My brother has an accident with a bike. I help him immediately. | Peer willingness | Negative Emotions ᵃ | Retained |
| 7 | The old lady next door is lying unconscious. I do not know what to do. | Negative Emotions | Negative Emotions | Retained |
| 8 | A homeless person is lying unconscious on the street. I help him immediately. | Stranger willingness | First Aid Willingness | Retained |
| 9 | Someone at school is lying on the floor. I walk there and ask how he is. | Peer willingness | First Aid Willingness | Retained |
| 10 | A woman lies unconscious in the corner. I know how to perform CPR. | Knowledge | Perceived Knowledge | Retained |
| 11 | Two of my classmates were injured before class. I ask for help immediately. | Peer willingness | First Aid Willingness | Retained |
| 12 | My brother falls while skating. I help him immediately. | Peer willingness | Negative Emotions ᵃ | Retained |
| 13 | Two unknown cyclists collide. I help them and ask for help if necessary. | Stranger willingness | First Aid Willingness | Retained |
| 14 | A boy fell out of the swing in the playground. I help him immediately. | Peer willingness | First Aid Willingness | Retained |
| 15 | In the case of a street accident, I know how to ask for medical help. | Knowledge | Perceived Knowledge | Retained |
| 16 | If I see someone injured, I help him immediately. | Stranger willingness | First Aid Willingness | Retained |
| 17 | I would be afraid to help someone who is unconscious. | Negative Emotions | — (removed) | Removed ᵇ |
| 18 | In the case of a serious accident in my neighbourhood, I would panic. | Negative Emotions | — (removed) | Removed ᶜ |

*ᵃ Source Items 6 and 12 were peer-directed willingness items in the source instrument but loaded on the Negative Emotions factor in the present Turkish sample (see Discussion). ᵇ Item 17 was removed because of an ambiguous cross-loading pattern. ᶜ Item 18 was removed because of low communality (0.14) after the removal of Item 17. Turkish and source item numbers are identical; the full 18-item pool of Katona, Tarkó, and Berki (2022) was translated.*

**Table S2. Item-level content validity (Lawshe's method, 12 experts)**

Content validity was evaluated by a 12-member expert panel (six professors [PhD] and three associate professors [PhD] in Pediatric Nursing, two professors [MD] in Pediatrics, and one associate professor in Biostatistics). Each item was rated for essentiality using Lawshe's three-category format, and the content validity ratio (CVR) was computed as CVR = (nₑ − N/2)/(N/2). For a 12-member panel, the critical CVR is 0.56.

All item-level CVRs exceeded 0.83, and the scale-level content validity index (S-CVI/Ave) was 0.90, both exceeding the recommended thresholds.

| **#** | **Academic title** | **Degree** | **Field of expertise** | **n** |
| --- | --- | --- | --- | --- |
| 1 | Professor | PhD | Pediatric Nursing (Child Health and Diseases Nursing) | 6 |
| 2 | Associate Professor | PhD | Pediatric Nursing (Child Health and Diseases Nursing) | 3 |
| 3 | Professor | MD | Pediatrics (Child Health and Diseases) | 2 |
| 4 | Associate Professor | PhD | Biostatistics | 1 |

*Note. The panel combined clinical and academic expertise in child health and pediatric nursing (11 experts) with methodological/statistical expertise (1 biostatistician), covering both the content domain and the measurement properties of the instrument. All 12 experts rated every item.*

**Table S3. Item-response distributions, skewness, and kurtosis (N = 470)**

| **Item** | **1** | **2** | **3** | **4** | **5** | **M** | **SD** | **Skew** | **Kurt** |
| --- | --- | --- | --- | --- | --- | --- | --- | --- | --- |
| I1 | 19 | 10 | 62 | 110 | 269 | 4.28 | 1.04 | -1.55 | 1.93 |
| I2 | 201 | 120 | 94 | 35 | 20 | 2.05 | 1.14 | 0.88 | -0.09 |
| I3 | 142 | 109 | 48 | 120 | 51 | 2.64 | 1.41 | 0.25 | -1.38 |
| I4 | 27 | 15 | 73 | 108 | 247 | 4.13 | 1.14 | -1.31 | 0.94 |
| I5 | 33 | 27 | 72 | 92 | 246 | 4.04 | 1.24 | -1.15 | 0.23 |
| I6 | 110 | 39 | 160 | 31 | 130 | 3.07 | 1.48 | -0.04 | -1.27 |
| I7 | 12 | 196 | 108 | 107 | 47 | 2.96 | 1.07 | 0.44 | -0.93 |
| I8 | 87 | 104 | 136 | 83 | 60 | 2.84 | 1.28 | 0.13 | -0.99 |
| I9 | 62 | 61 | 97 | 112 | 138 | 3.43 | 1.37 | -0.43 | -1.04 |
| I10 | 315 | 66 | 49 | 20 | 20 | 1.65 | 1.10 | 1.69 | 1.92 |
| I11 | 31 | 22 | 67 | 116 | 234 | 4.06 | 1.19 | -1.22 | 0.58 |
| I12 | 105 | 136 | 71 | 142 | 16 | 2.63 | 1.22 | 0.10 | -1.31 |
| I13 | 48 | 54 | 108 | 112 | 148 | 3.55 | 1.31 | -0.52 | -0.85 |
| I14 | 42 | 41 | 91 | 100 | 196 | 3.78 | 1.31 | -0.78 | -0.56 |
| I15 | 106 | 84 | 102 | 79 | 99 | 2.96 | 1.45 | 0.04 | -1.33 |
| I16 | 33 | 55 | 122 | 114 | 146 | 3.61 | 1.23 | -0.50 | -0.73 |
| I17 | 78 | 86 | 118 | 77 | 111 | 3.12 | 1.39 | -0.06 | -1.23 |
| I18 | 104 | 89 | 109 | 72 | 96 | 2.93 | 1.43 | 0.09 | -1.29 |

*Note. Columns 1–5 give the frequency of each response category. All items used the full 1–5 range. No item showed floor or ceiling concentration precluding ordinal factor analysis. Analytic sample comprised complete cases with no missing data.*

**Table S4. Initial 18-item EFA pattern matrix (PAF, Promax, polychoric)**

| **Item** | **F1** | **F2** | **F3** | **F4** | **h²** |
| --- | --- | --- | --- | --- | --- |
| I1 | 0.734 | -0.058 | -0.245 | -0.055 | 0.467 |
| I2 | -0.002 | -0.062 | 0.778 | -0.010 | 0.591 |
| I3 | -0.007 | 0.645 | -0.026 | -0.060 | 0.413 |
| I4 | 0.722 | -0.069 | 0.072 | -0.042 | 0.547 |
| I5 | 0.611 | -0.083 | -0.085 | -0.157 | 0.346 |
| I6 | 0.053 | 0.663 | -0.028 | -0.013 | 0.447 |
| I7 | -0.013 | 0.642 | 0.007 | 0.020 | 0.411 |
| I8 | 0.572 | 0.064 | 0.158 | -0.016 | 0.433 |
| I9 | 0.564 | 0.142 | 0.057 | 0.163 | 0.425 |
| I10 | -0.160 | -0.039 | 0.885 | 0.056 | 0.686 |
| I11 | 0.788 | 0.006 | -0.086 | 0.022 | 0.587 |
| I12 | -0.073 | 0.846 | -0.044 | -0.011 | 0.690 |
| I13 | 0.783 | 0.060 | 0.003 | 0.076 | 0.646 |
| I14 | 0.743 | -0.034 | 0.068 | 0.165 | 0.622 |
| I15 | 0.185 | 0.024 | 0.629 | -0.123 | 0.559 |
| I16 | 0.738 | -0.003 | 0.111 | -0.019 | 0.611 |
| I17 | -0.018 | -0.308 | 0.316 | 0.401 | 0.426 |
| I18 | 0.059 | 0.001 | 0.101 | 0.846 | 0.707 |

*Note. Principal axis factoring of the polychoric correlation matrix with Promax rotation; four factors extracted (parallel-analysis suggestion). Loadings ≥ |0.40| indicate the primary factor. Items 17 and 18 form a weak fourth factor (F4). h² = communality.*

**Table S5. Intermediate 17-item EFA pattern matrix (after removing Item 17)**

| **Item** | **F1** | **F2** | **F3** | **F4** | **h²** |
| --- | --- | --- | --- | --- | --- |
| I1 | 0.891 | -0.020 | -0.257 | -0.193 | 0.563 |
| I2 | -0.015 | -0.051 | 0.802 | -0.106 | 0.591 |
| I3 | 0.025 | 0.660 | -0.011 | -0.096 | 0.422 |
| I4 | 0.688 | -0.057 | 0.103 | 0.020 | 0.543 |
| I5 | 0.779 | -0.042 | -0.059 | -0.283 | 0.434 |
| I6 | 0.027 | 0.665 | -0.019 | 0.012 | 0.447 |
| I7 | -0.031 | 0.644 | 0.012 | 0.002 | 0.413 |
| I8 | 0.471 | 0.062 | 0.193 | 0.113 | 0.431 |
| I9 | 0.327 | 0.107 | 0.041 | 0.433 | 0.502 |
| I10 | -0.196 | -0.035 | 0.875 | -0.047 | 0.648 |
| I11 | 0.768 | 0.017 | -0.084 | 0.051 | 0.591 |
| I12 | -0.083 | 0.844 | -0.042 | -0.012 | 0.684 |
| I13 | 0.593 | 0.037 | 0.007 | 0.332 | 0.680 |
| I14 | 0.533 | -0.066 | 0.054 | 0.391 | 0.668 |
| I15 | 0.189 | 0.045 | 0.698 | -0.165 | 0.581 |
| I16 | 0.657 | 0.000 | 0.143 | 0.092 | 0.603 |
| I18 | -0.047 | -0.042 | -0.100 | 0.417 | 0.112 |

*Note. After the removal of Item 17, Item 18 retained a communality of only 0.14, indicating minimal shared variance with the remaining items; it was therefore removed in the next step. The final 16-item three-factor structure is reported in the main text.*

**Table S6. Standardized loadings of the final 16-item three-factor CFA (WLSMV)**

| **Factor** | **Item** | **Std. loading** |
| --- | --- | --- |
| First Aid Willingness | I13 | 0.808 |
|  | I11 | 0.741 |
|  | I14 | 0.784 |
|  | I16 | 0.783 |
|  | I1 | 0.613 |
|  | I4 | 0.734 |
|  | I9 | 0.638 |
|  | I5 | 0.540 |
|  | I8 | 0.666 |
| Negative Emotions | I12 | 0.788 |
|  | I6 | 0.687 |
|  | I7 | 0.655 |
|  | I3 | 0.654 |
| Perceived Knowledge | I10 | 0.675 |
|  | I2 | 0.720 |
|  | I15 | 0.892 |

*Note. Scaled χ²(101) = 288.09, p < .001; CFI = 0.952; TLI = 0.943; RMSEA = 0.063, 90% CI [0.054, 0.071]; SRMR = 0.061. Composite reliability: 0.90 (FAW), 0.79 (NE), 0.81 (PK); AVE: 0.50, 0.49, 0.59, respectively.*

**Final 16-item Turkish FAWQ (FAWQ-TR)**

Response scale: 1 = “Hiç bana göre değil” (not at all typical of me) … 5 = “Tamamen bana göre” (completely typical of me).

| **#** | **Madde (Turkish item)** | **Subscale** |
| --- | --- | --- |
| 1 | Arkadaşım oyun alanında yaralandığında ona hemen yardım ederim | First Aid Willingness |
| 2 | Kanayan bir baş gördüğümde basınçlı bandajın nasıl uygulanacağını bilirim | Perceived Knowledge |
| 3 | Sınıf arkadaşım parmağını kestiğinde ve parmağı kanadığında endişelenmeye başlarım | Negative Emotions |
| 4 | Yaşlı bir adamın sokakta bayıldığını fark ettiğimde yardım çağırırım | First Aid Willingness |
| 5 | Kuzenim kolunu kırdığında ona hemen yardım ederim | First Aid Willingness |
| 6 | Kardeşim bisiklet kazası geçirdiğinde ona hemen yardım ederim | Negative Emotions |
| 7 | Komşumuz yaşlı kadın baygın bir şekilde yerde yattığında ne yapacağımı bilemem | Negative Emotions |
| 8 | Evsiz bir kişi sokakta baygın yattığında ona hemen yardım ederim | First Aid Willingness |
| 9 | Okuldan biri yerde uzandığında oraya gidip nasıl olduğunu sorarım | First Aid Willingness |
| 10 | Bir kadın köşede baygın yattığında CPR'nin nasıl yapılacağını bilirim | Perceived Knowledge |
| 11 | Sınıf arkadaşlarımdan ikisi dersten önce yaralandığında hemen yardım isterim | First Aid Willingness |
| 12 | Kardeşim paten yaparken düştüğünde ona hemen yardım ederim | Negative Emotions |
| 13 | Tanımadığım iki bisikletli çarpıştığında onlara yardım eder ve gerekirse yardım isterim | First Aid Willingness |
| 14 | Oyun alanında bir çocuk salıncaktan düştüğünde hemen yardım ederim | First Aid Willingness |
| 15 | Sokakta kaza olduğu durumda nasıl tıbbi yardım isteneceğini bilirim | Perceived Knowledge |
| 16 | Yaralı birini görsem hemen yardım ederim | First Aid Willingness |

*Item numbers follow the original source numbering. Items 17 and 18 were removed and are not part of the final scale. Permission to translate, adapt, and use the instrument was obtained from the developer.*

**Scoring guide**

**Subscales (mean of items, 1–5 metric)**

• First Aid Willingness (FAW): items 1, 4, 5, 8, 9, 11, 13, 14, 16 - 9 items.

• Negative Emotions (NE): items 3, 6, 7, 12 - 4 items.

• Perceived Knowledge (PK): items 2, 10, 15 - 3 items.

**Direction and interpretation**

Each subscale score is the arithmetic mean of its constituent items on the original 1–5 metric. The Negative Emotions items are scored in the direction of the construct, so that higher scores indicate stronger anticipated negative emotions (e.g., fear or panic); they are not reverse scored, because the three subscales are interpreted independently and no composite total score combining the domains is computed. Higher First Aid Willingness and Perceived Knowledge scores denote greater readiness and self-assessed capability, whereas higher Negative Emotions scores denote greater affective barriers to intervention.

No overall FAWQ total score is recommended, because a general or higher-order factor was not evaluated. Missing responses: the analytic sample consisted of complete cases; if missing data occur in future applications, subscale means may be computed when at least two-thirds of a subscale's items are answered.

**Reliability (present sample)**

Cronbach's α / McDonald's ω: FAW = 0.87 / 0.87; NE = 0.73 / 0.75; PK = 0.72 / 0.74.
