# Supplementary material for: Cross-cultural adaptation, validation, and measurement invariance of the First Aid Willingness Questionnaire (FAWQ) among Turkish adolescents
Source: PLoS One. 2026 Oct 23;21(10):e0361329. doi: 10.1371/journal.pone.0361329 (PMC13693896; doi:10.1371/journal.pone.0361329)
Supplement: Supplementary file 2 [file pone.0361329.s003.docx]

**BİLGİLENDİRİLMİŞ GÖNÜLLÜ ONAM FORMU (EK-3)**

Sayın Anne/Baba

Sizi Aksaray/Merkez’de yürütülen **“Okul Çocukları İçin İlk Yardım İstekliliği Anketi Türkçe Formunun Psikometrik Özelliklerinin İncelenmesi”** başlıklı araştırmaya davet ediyoruz. Bu araştırmaya katılıp katılmama kararını vermeden önce, araştırmanın ne amaçla ve nasıl yapılacağını, bu araştırmanın gönüllü katılımcılara getireceği olası faydaları, riskleri ve rahatsızlıklarını bilmeniz ve kararınızı bu bilgilendirme çerçevesinde özgürce vermeniz gerekmektedir. Bu nedenle bu formun okunup anlaşılması büyük önem taşımaktadır. Bu form araştırma sorumlusu olarak bizler tarafından size sözel olarak aktarılan bilgilendirmenin yazılı şeklini içermektedir. Formu imzalamadan önce size sözel olarak da anlatılan aşağıdaki bilgileri birkez de dikkatlice okumak için zaman ayırınız. Katılmayı kabul ettiğiniz takdirde, tarafınız ve bilgilendirme esnasında yanınızda olan tanık kişi tarafından imzalanan bu formun bir kopyası saklamanız için size verilecektir.

Araştırmaya katılmak tamamen gönüllülük esasına dayanmaktadır. Çalışmaya katılmama veya katıldıktan sonra herhangi bir anda çalışmadan çıkma hakkına sahipsiniz. İstemediğiniz sorulara cevap vermeme hakkına sahipsiniz. Her üç durumda da hiçbir yaptırıma ve hak kaybına maruz kalmayacağınızı bildirmek isteriz.

Ayrıca yapılacak olan çalışmada / araştırmada “Kişisel Verilerin Korunması Kanununun” ilgili maddeleri dikkate alınacağını belirtmek isteriz.

***Araştırma Sorumlusu***

***(Özlem ÇALIŞIR***)

**Araştırmanın Amacı**

Gönüllü olarak katılmanızı teklif ettiğimiz çalışma bir araştırma projesidir. Bu araştırma projesinin amacı; Okul Çocukları İçin İlk Yardım İstekliliği Anketi’nin Türkçe’ ye adaptasyonu ve ölçeğin psikometrik özelliklerinin doğrulanmasıdır.

**Araştırmaya Dair Araştırmacı ve Katılımcı Hakkında Bilgiler**

Araştırma, 2024-2025 eğitim öğretim yılında Aksaray ili İl Milli Eğitim Müdürlüğü’ne bağlı merkez ilçelerdeki ortaöğretim kurumlarında yapılacaktır. Araştırmanın evrenini Aksaray ilindeki ortaöğretim (lise) kurumlarında öğrenim gören 16.229 (devlet+özel) çocuk oluşturmaktadır. Ölçek uyarlama çalışmalarında örnekleme alınacak birey sayısının ölçekteki madde sayısının yaklaşık 5-20 katı olması önerilmektedir (DeVellis, 2017). Fakat faktörlerdeki madde sayısı az, faktör yükleri orta ve düşük olduğu zaman örneklem büyüklüğünün en az 300 olması önerilmektedir (Veneziano L, Hooper J. 1997). Ayrıca doğrulayıcı faktör analizi için ise önerilen örneklem büyüklüğü 300-500 olarak belirtilmektedir (Sousa VD ve Rojjanasrirat W. 2011). Bu nedenle araştırma örneklemine ölçekteki madde sayısının 20 katı kadar birey alınması planlanmıştır (18x20=360). Veri kaybı olabileceği göz önüne alınarak araştırmaya katılmayı kabul eden 13-16 yaş arası 500 ergenin araştırma kapsamına alınması planlanmıştır. Araştırmaya katılmaya ailesi izin veren ve gönüllü olan lise öğrencileri, kaynaştırma öğrencisi olmayan, bedensel, zihinsel bir engel ve uzuv kaybı olmayan, Türkçe’ yi anlayıp, konuşup, okuyabilen ve yazabilen 13-16 yaş arası olan öğrenciler araştırmaya dâhil edilecektir. Araştırma yaklaşık 15 dakika sürecektir.

**Araştırmanın Size Getirebileceği Risk ve Rahatsızlıklar Nelerdir?**

Araştırmanın getireceği risk yoktur.

**Araştırmanın Topluma ve Size Olası Faydaları**

Ülkemizde okul çağındaki öğrencilerin ilk yardım istekliliğini ölçebilecek bir araç geliştirerek bu alanda yapılacak çalışmalara ışık tutulacaktır.

**Araştırmaya Katılma / Ayrılma Konusunda Haklarınız ve Araştırmacının Haklarınızı Koruma Güvencesi**

Bu araştırmada yer almak tamamen sizin isteğinize bağlıdır. Araştırmada yer almayı reddedebilirsiniz ya da başladıktan sonra herhangi bir zamanda bırakabilirsiniz. Çalışmaya katılmama, çalışmadan çıkma veya çıkarılma durumlarında herhangi bir ceza ya da yararınıza olan hakların kaybı kesinlikle söz konusu olmayacaktır. Araştırma konusu ile ilgili araştırmaya devam etme isteğinizi etkileyebilecek yeni bilgiler elde edilmesi durumunda siz ya da yasal temsilciniz bilgilendirilecektir.

Araştırmanın sonuçları bilimsel ve eğitim amaçları ile kullanılacaktır. Sizden elde edilen tüm bilgiler tamamen araştırma amacı ile kullanılacak, gizli tutulacak, araştırma yayınlandığında da varsa kimlik bilgilerinizin gizliliği korunacaktır.

**İletişim Kurulacak Kişi(ler)**

*Özlem ÇALIŞIR*

*TEL: 0531 706 13 56*

**Yukarıda Belirtilen Hususlar Dışında Sorularınız Var İse, Bu Bölüme Eklenerek Cevaplandırılacaktır.**

☐**Gönüllünün sorduğu ek sorular ve cevapları** ☐ **Ek bir soru bulunmamaktadır.**

**RIZA / ONAY / ONAM**

Yukarıda konusu ve amacı belirtilen araştırmaya ilişkin bilgilendirme bölümünü okudum ve aşağıda imzası olan ilgili tarafından önce sözlü sonra yazılı olarak bilgilendirildim. Katılmam istenen çalışmanın kapsamını ve amacını, gönüllü olarak üzerime düşen sorumlulukları tamamen anladım. **Çalışma hakkında soru sorma ve tartışma imkânı buldum ve tatmin edici yanıtlar aldım. Bana, çalışmanın muhtemel riskleri ve faydaları sözlü olarak da anlatıldı.** Araştırmaya gönüllü olarak katıldığımı, istediğim zaman gerekçeli veya gerekçesiz olarak araştırmadan ayrılabileceğimi ve kendi isteğime bakılmaksızın araştırmacı tarafından araştırma dışı bırakılabileceğimi biliyorum.

Bu koşullarda;

1) Söz konusu araştırmaya hiçbir baskı ve zorlama olmaksızın kendi rızamla katılmayı (çocuğumun/vasimin bu çalışmaya katılmasını) kabul ediyorum.
2) Gerek duyulursa kişisel bilgilerime mevzuatta belirtilen kişi/kurum/kuruluşların erişebilmesine,
3) Çalışmada elde edilen bigilerin (*kimlik bilgilerim gizli kalmak koşulu ile*) yayın için kullanılma, arşivleme ve eğer gerek duyulursa bilimsel katkı amacı ile ülkemiz dışına aktarılmasına olur veriyorum.

Ek başkaca bir açıklamaya gerek duymadan, hiçbir baskı altında kalmadan ve bilinçli olarak bu araştırmaya katılmayı onaylıyorum

| ***Gönüllünün (Kendi el yazısı ile)***  Adı-Soyadı:  İletişim  Tarih:  İmzası: | ***Velayet veya Vesayet Altında Bulunanlar İçin*** ***Veli veya Vasisinin*** (kendi el yazısı ile)  Adı-Soyadı:  İletişim:  Tarih:  İmzası: |
| --- | --- |
|  |  |
|  |  |

Yukarıda ismi yazılı gönüllüye / yasal temsilcisine tarafımdan araştırmanın amacı, içeriği, yöntemi, fayda ve riskleri, gönüllüye ait haklar konusunda açıklamalar yapılmıştır. Ayrıca gönüllünün / yasal temsilcisinin işbu formu ayrıntılı inceleyerek imzalaması sağlanmıştır.

Açıklamaları Yapan Kişinin	Adı-Soyadı: ÖZLEM ÇALIŞIR

**ÖĞRENCİ TANIMA FORMU (EK-1)**

1. **Cinsiyeti:** Erkek ( )	Kız ( )
2. **Yaşı:** ………….
3. **Sınıfı**: ………...
4. **Anne Yaşı:** …………….
5. **Annenin Çalışma Durumu**:

Çalışıyor ( ) ……………… Çalışmıyor ( )

6. **Anne Eğitim Durumu:** ( ) Okuryazar, ancak bir okul bitirmedi ( ) İlköğretim mezunu

( ) Lise mezunu	( ) Üniversite mezunu

( )Diğer……

7. **Baba Yaşı:** ……………..
8. **Babanın Çalışma Durumu:**

Çalışıyor ( ) …………….. Çalışmıyor ( )

9. **Baba Eğitim Durumu:** ( ) Okur yazar, ancak bir okul bitirmedi ( ) İlköğretim mezunu

( ) Lise mezunu	( )Üniversite mezunu

( ) Diğer………..

10. **Aile Tipi**

( ) Çekirdek aile (anne, baba, çocuklar)

( )Geniş aile (anne, baba, çocuklar ve aile büyükleri)

( )Parçalanmış aile (boşanma, ayrı yaşama ya da ölüm vb. )

**11. Ailenin Ekonomik Durumu (öğrencinin ifadesine göre)**

12. **Daha önce bir yaralıya müdahale ettiniz mi**? ( ) Evet …………….( )Hayır
13. **Daha önce ilk yardım ile ilgili bilgi/eğitim aldınız mı?** ( )Evet ……..( )Hayır
14. **İlk yardım bilmek sizce gerekli midir?** ( ) Evet………….( ) Hayır
15. **İlkyardım eğitimi ilkokuldan itibaren verilmeli midir?**

( ) Evet……………………….( ) Hayır

16. **İlk yardım gibi sağlıkla ilgili konuları nereden öğrenmek istersiniz?**

( ) Aileden

( ) Öğretmenden

( ) İnternetten-Televizyondan

( ) Teknolojik araç-gereçlerden

( ) Gazete, dergi

( ) Ders kitabından

( ) Sağlık personelinden

( ) diğer…………..

( ) Gelir giderden az

( ) Gelir gidere eşit

( ) Gelir giderden fazla

**İlk Yardım Gönüllülük Anketi (İYGA)**

1: Hiç bana göre değil <==> 5: Tamamen bana göre

|  | **Maddeler** | **1** | **2** | **3** | **4** | **5** |
| --- | --- | --- | --- | --- | --- | --- |
| 1. | Arkadaşım oyun alanında yaralandığında ona hemen yardım ederim |  |  |  |  |  |
| 2. | Kanayan bir baş gördüğümde basınçlı bandajın nasıl uygulanacağını bilirim |  |  |  |  |  |
| 3. | Sınıf arkadaşım parmağını kestiğinde ve parmağı kanadığında endişelenmeye başlarım |  |  |  |  |  |
| 4. | Yaşlı bir adamın sokakta bayıldığını fark ettiğimde yardım çağırırım |  |  |  |  |  |
| 5. | Kuzenim kolunu kırdığında ona hemen yardım ederim |  |  |  |  |  |
| 6. | Kardeşim bisiklet kazası geçirdiğinde ona hemen yardım ederim |  |  |  |  |  |
| 7. | Komşumuz yaşlı kadın baygın bir şekilde yerde yattığında ne yapacağımı bilemem |  |  |  |  |  |
| 8. | Evsiz bir kişi sokakta baygın yattığında ona hemen yardım ederim |  |  |  |  |  |
| 9. | Okuldan biri yerde uzandığında oraya gidip nasıl olduğunu sorarım |  |  |  |  |  |
| 10. | Bir kadın köşede baygın yattığında KPR' nin nasıl yapılanacağını bilirim |  |  |  |  |  |
| 11. | Sınıf arkadaşlarımdan ikisi dersten önce yaralandığında hemen yardım isterim |  |  |  |  |  |
| 12. | Kardeşim paten yaparken düştüğünde ona hemen yardım ederim |  |  |  |  |  |
| 13. | Tanımadığım iki bisikletli çarpıştığında onlara yardım eder ve gerekirse yardım isterim |  |  |  |  |  |
| 14. | Oyun alanında bir çocuk salıncaktan düştüğünde hemen yardım ederim |  |  |  |  |  |
| 15. | Sokakta kaza olduğu durumda nasıl tıbbi yardım isteneceğini bilirim |  |  |  |  |  |
| 16. | Yaralı birini görsem hemen yardım ederim |  |  |  |  |  |
| 17. | Bilinci yerinde olmayan birine yardım etmekten korkarım |  |  |  |  |  |
| 18. | Mahallemde ciddi bir kaza olduğunda paniğe kapılırım |  |  |  |  |  |
